# Association of *PIK3CA* and *MDM2* SNP309 with Cervical Squamous Cell Carcinoma in a Philippine Population

**DOI:** 10.31557/APJCP.2019.20.7.2103

**Published:** 2019

**Authors:** Ourlad Alzeus G Tantengco, Yukiko Nakura, Michinobu Yoshimura, Erlidia F Llamas-Clark, Itaru Yanagihara

**Affiliations:** 1 *College of Medicine, University of the Philippines Manila,*; 3 *Department of Obstetrics and Gynecology, College of Medicine, University of the Philippines-Philippines General Hospital, Manila, Philippines*; 2 *Department of Developmental Medicine, Research Institute, Osaka Women's and Children's Hospital, Izumi, Osaka, Japan.*

**Keywords:** *MDM2*- *PIK3CA*- mutation- HPV- cervical cancer

## Abstract

**Background:**

*PIK3CA* and *MDM2* SNP309 have been studied to be associated with cervical cancer. PIK3CA mutation is associated with poor treatment response and low survival rate while *MDM2* is associated with tumorigenesis and poor prognosis in cervical cancer. Thus, we determined the prevalence of *PIK3CA* and *MDM2* mutations in Filipino cervical cancer patients.

**Methods:**

Twenty-eight formalin-fixed paraffin-embedded cervical squamous cell carcinoma and 16 non-malignant cervix tissue biopsies of Filipino patients were subjected to *PIK3CA* gene and *MDM2* SNP309 (*rs2279744*) analysis.

**Results:**

*PIK3CA* gene was found mutated in three (10.71 %) out of 28 cervical cancer patients included in this study. Among the HPV-negative cervical cancer patients, two (28.57 %) were positive for *PIK3CA* mutation and only one (4.76 %) tested positive among the HPV-positive cervical cancer patients. *MDM2* SNP309 analysis revealed that T allele (71.43%) was more common in cervical cancer patients compared to the control group. TG genotype (p = 0.03; OR = 0.18, 95% CI 0.04-0.76) was associated with lower rates of cervical cancer when TT genotype was used as a reference point.

**Conclusion:**

*PIK3CA* gene mutation was present among Filipino cervical cancer patients and not in control patients. *MDM2* SNP309 analysis revealed that TG genotype has lower association to cervical cancer when compared with the TT and GG genotypes.

## Introduction

Cervical cancer continues to be the second most frequent cancer among Filipino women. It is estimated that almost 6,670 Filipino women are diagnosed with cervical cancer and 2,832 die from this disease annually (ICO/IARC Information Centre on HPV and Cancer, 2017). One of the established necessary risk factors for cervical cancer are oncogenic types of human papillomavirus (HPV). Cells infected by this virus can develop precancerous properties which can lead to cervical intraepithelial neoplasia (CIN) or adenocarcinoma in situ (AIS). Without treatment, CIN grade II/III and AIS can progress to squamous cell carcinoma or adenocarcinoma, respectively (Bosch et al., 1995).

Nucleic acid sequencing is now being used to determine gene mutations that can be used as potential diagnostic or prognostic tools for cancer patients. In cervical cancer patients, mutations in *PIK3CA* gene was found to be associated with worse overall survival and cancer-specific survival (Lachkar et al., 2018). A study in Chinese population showed that patients with *PIK3CA* mutations belong to older populations and postmenopausal patients. It is more common among patients with cervical squamous cell carcinoma and is associated with distant metastases (Xiang et al., 2015). Another study detected *PIK3CA* gene mutation rate of 11% in adenocarcinoma, and 5% in squamous cell carcinoma patients (Tornesello et al., 2014).

Murine double-minute 2 homolog (*MDM2*) is another gene that has been studied to be associated with tumorigenesis and poor prognosis in several types of cancer including cervical cancer (Zhang et al., 2017). *MDM2 *single nucleotide polymorphism gene (*rs2279744*) is in the position 309 in the first intron of the *MDM2 *oncogene. Replacement of T with G allele increased *MDM2 *mRNA and protein synthesis and decreased the p53 tumor suppressor activity. *MDM2 *SNP309 (*rs2279744*) was found to be associated in the development of cervical lesions in women with HPV infection. It might also serve as a marker for assessing the progression low squamous intraepithelial lesion (LSIL) to high squamous intraepithelial lesion (HSIL) (Amaral et al., 2014). 

Identification of known genetic mutations and polymorphisms in cervical cancer and their association with HPV infection status will help in further understanding the contribution of somatic genomic alteration in the pathogenesis of cervical cancer. Therefore, in this study, we investigated the frequency of *PIK3CA* and *MDM2 *mutations in Filipino patients with cervical squamous cell carcinoma.

## Materials and Methods

*Patient and tissue samples*

This is a case-control study which included 28 formalin-fixed paraffin-embedded cervical squamous cell carcinoma and 16 non-malignant cervix tissue biopsies of patients seen at the Philippine General Hospital from January to December 2017. Cases and control samples were selected through simple random sampling from the list of cervical cancer patients during 2017 in the Philippine General Hospital. Patients diagnosed with cervical squamous cell carcinoma and with complete clinical data were included in the study. Exclusion criteria include antimicrobial treatment within one month prior to the present study. Matching was not performed in this study. This study was approved by the University of the Philippines Manila Research Ethics Board (UPMREB Registration No.: 2018-016-01).

*DNA Extraction*

De-identified formalin fixed paraffin embedded (FFPE) cervical tissues were retrieved from the Section of Surgical Pathology at the Department of Laboratories of the Philippine General Hospital. Approximately, 5 μm slices of the FFPE samples were used for total genomic DNA extraction according the protocol of Maxwell^® ^RSC DNA FFPE Kit (Promega, Japan). The concentration of the extracted DNA in aqueous solution was checked using NanoDrop 2000 Spectrophotometer (Thermo Fisher Scientific, Wilmington, DE, USA).

*PIK3CA exon 9 mutation analysis*

*PIK3CA* exon 9 was amplified by PCR using the primers: PIK3-9-F3 (5′-GAGTAACAGACTAGCTAGAGAC-3′) with PIK3-9-R2 (5′-ACAGAGAATCTCCATTTTAGC-3′). PCR reactions were performed using 1 μL gDNA, 6.25 μL 2 × QuantiTect^®^ SYBR^®^ Green PCR Master Mix (Qiagen, Japan), 0.625 μL each of 10 μM primers and 4 μL of deionized distilled water in a total volume of 12.5 μL. The PCR assay was performed using MJ Research, Chromo 4 system (Reno, Nevada, USA). The PCR was conducted with an initial denaturation of 95°C for 15 min, followed by 35 cycles of 95°C for 20 sec, annealing temperature of 58°C for 30 sec, extension temperature of 72°C for 30 sec, and a final cycle of 72°C for 4 min. 

*MDM2 SNP309 (rs2279744) analysis*

*MDM2 *gene including the SNP309 (*rs2279744*) region was amplified using the following primer sequences: forward (5′-CGGGAGTTCAGGGTAAAGGT-3′) and reverse (5′-AGCAAGTCGGTGCTTACCTG-3′) (Hu et al., 2010). PCR reactions were performed using 1 μL gDNA, 6.25 μL 2 × QuantiTect^®^ SYBR^®^ Green PCR Master Mix (Qiagen, Japan), 0.625 μL each of 10 μM primers and 4 μL of deionized distilled water in a total volume of 12.5 μL. The assays were performed using MJ Research, Chromo 4 system (Reno, Nevada, USA). The PCR was conducted with an initial denaturation of 95°C for 15 min, followed by 35 cycles of 95°C for 20 sec, annealing temperature of 63°C for 30 sec, extension temperature of 72°C for 30 sec, and a final cycle of 72 °C for 4 min. 

*Nucleic acid sequencing*

All PCR amplicons of *PIK3CA* and *MDM2 *genes were purified using NucleoSpin^®^ Gel and PCR Clean Up (Machery-Nagel, Germany) and were subjected to DNA nucleotide sequencing using the Big Dye™ Terminator v3.1 Cycle Sequencing Kit on an ABI 3130 Genetic Analyzer (Thermo Fisher Scientific, MA, 220 USA) according to the manufacturer’s instructions.

*Statistical Analysis*

Fisher exact test and t-test were calculated using Open Epi Software (https://www.openepi.com/Menu/OE_Menu.htm). Allele frequencies were determined by the direct count of the alleles. Genotypic distributions were examined for significant departure from Hardy–Weinberg equilibrium by Chi square-test. SNPStat online software (https://www.snpstats.net/start.htm) was used to analyze the association of the allele and genotype frequencies with cervical cancer. Association was considered statistically significant when p value was less than 0.05.

## Results

This study included 28 cases of cervical cancer and 21 controls with non-malignant cervix from the Philippine General Hospital. The mean age of the cervical cancer patients was 52.75 ± 13.16 years while the control patients was 47.31 ± 15.02 years. Patients in the case group were significantly older compared to patients in the control group.

**Table 1:** Histological Characteristics of Cervical Cancer Samples, HPV status, PIK3CA (exon 9) Mutations and *MDM2* Single Nucleotide Polymorphisms

Patient No	Histology	FIGO Staging	HPV Status	*PIK3CA* mutation	*MDM2* SNP309
p1	Cervical SCCA, nonkeratinizing	3B	Negative		T/T
p2	Cervical SCCA, nonkeratinizing	3B	HPV 16		T/G
p3	Cervical SCCA, nonkeratinizing	3B	HPV 16		T/T
p4	Cervical SCCA, nonkeratinizing	3B	HPV 16, 18		T/T
p5	Cervical SCCA, nonkeratinizing	3B	Negative		T/T
p6	Cervical SCCA, nonkeratinizing	3B	HPV 16		T/T
p7	Cervical SCCA, nonkeratinizing	4A	HPV 52		T/T
p8	Cervical SCCA, nonkeratinizing	3B	HPV 18		T/G
p9	Cervical SCCA, nonkeratinizing	2B	HPV 16, 52		G/G
p10	Cervical SCCA, nonkeratinizing	3B	HPV 16, 18		T/T
p11	Cervical SCCA, nonkeratinizing	3B	HPV 16		T/T
p12	Cervical SCCA, nonkeratinizing	3B	HPV 52		T/G
p13	Cervical SCCA, nonkeratinizing	3B	Negative		T/T
p14	Cervical SCCA, nonkeratinizing	3B	HPV 52		T/G
p15	Cervical SCCA, nonkeratinizing	2B	HPV 16		T/T
p16	Cervical SCCA, nonkeratinizing	3B	HPV 18	E545K	T/T
p17	Cervical SCCA, nonkeratinizing	3A	HPV 16		T/T
p18	Cervical SCCA, nonkeratinizing	3B	HPV 52		TG
p19	Cervical SCCA, nonkeratinizing	3B	Negative	E545K	G/G
p20	Cervical SCCA, nonkeratinizing	3B	Negative	E545A	G/G
p21	Cervical SCCA, nonkeratinizing	3B	HPV 52		T/G
p22	Cervical SCCA, nonkeratinizing	3B	Negative		G/G
p23	Cervical SCCA, nonkeratinizing	3B	HPV 18		T/T
p24	Cervical SCCA, nonkeratinizing	3B	HPV 16		T/T
p25	Cervical SCCA, nonkeratinizing	1B1	HPV 16		T/T
p26	Cervical SCCA, nonkeratinizing	3B	HPV 18		T/T
p27	Cervical SCCA, nonkeratinizing	3B	Negative		T/G
p28	Cervical SCCA, nonkeratinizing	3B	HPV 16		T/G

**Table 2:** PIK3CA Mutation and Clinical Characteristics of Cervical Squamous Cell Carcinoma Patients (n=28)

	PIK3CA wild type (n=25)	PIK3CA mutant (n=3)	p values
Mean age ± SD	51.33 ± 14.46	48.33 ± 11.72	p = 0.71^*^
FIGO Staging			p > 0.99^†^
I (n=1)	1	0	
II (n=2)	2	0	
III (n=24)	21	3	
IV (n=1)	1	0	
HPV Status			p = 0.29^†^
Negative (n=7)	5 (71.43%)	2 (28.57%)	
Positive (n=21)	20 (95.24%)	1 (4.76%)	

* p-value from two sample independent t-test; †p value from Fisher exact test.

**Table 3:** HPV Status and Allele Frequency of *MDM2* SNP309 (*rs2279744*) in Patients with Cervical Cancer (n=28) and Normal Cervix (n=16)

HPV Status	Alleles	Cases	Control	OR (95% CI)	p values
HPV Positive Samples	T	33 (79.00%)	4 (50.00%)	1.00	0.10
	G	9 (21.00%)	4 (50.00%)	0.27 (0.06-1.31)	
HPV Negative Samples	T	7 (50.00%)	15 (62.00%)	1.00	0.45
	G	7 (50.00%)	9 (38.00%)	1.67 (0.44-6.33)	
All Samples	T	40 (71.43%)	19 (59.38%)	1.00	0.25
	G	16 (28.57%)	13 (40.62%)	0.58 (0.23-1.46)	

**Table 4:** HPV Status and Genotype Frequency of *MDM2* SNP309 (*rs2279744*) in Patients with Cervical Cancer (n=28) and Normal Cervix (n=16)

HPV Status	Genotypes	Cases	Control	OR (95% CI)	p values
HPV Positive Samples	TT	13 (61.90%)	0 (0%)	1.00	P = 0.02
	TG	7 (33.30%)	4 (100%)	0.00	
	GG	1 (4.80%)	0 (0%)	1.00	
HPV Negative Samples	TT	3 (42.90%)	4 (33.33%)	1.00	P = 0.09
	TG	1 (14.30%)	7 (58.33%)	0.19 (0.01 - 2.50)	
	GG	3 (42.90%)	1 (8.33%)	4.00 (0.27 - 60.33)	
All Samples	TT	16 (57.14%)	4 (25.00%)	1.00	p = 0.03
	TG	8 (28.57%)	11 (68.75%)	0.18 (0.04 - 0.76)	
	GG	4 (14.29%)	1 (6.25%)	1.00 (0.09 - 11.59)	

*PIK3CA gene mutation analysis*

All cervical cancer patients were analyzed for *PIK3CA* mutation (Table 1). Based on our previous study, 21 cervical cancer were HPV-positive and 7 were HPV-negative. The exon 9 of *PIK3CA* gene was found mutated in three (10.71%) out of 28 cervical cancer patients included in this study (Table 2). Two patients have *E545K* mutation while one has *E545A* mutation. All three *PIK3CA* gene mutants were from patients with FIGO stage 3 cervical squamous cell carcinoma. Among HPV-negative cervical cancer patients, two (28.57%) were positive for *PIK3CA* mutation while among HPV-positive cervical cancer patients, only one (4.76 %) tested positive for *PIK3CA* mutation. This mutation was not found among the control patients.

*MDM2 SNP309 (rs2279744) analysis*

The frequencies of each allele and genotype were shown in Table 3. *MDM2 *polymorphism analysis revealed that T allele (71.43%) was more common in cervical cancer patients compared to the control group. The G allele for *MDM2 *SNP309 did not show significant association with cervical cancer. The SNP was evaluated in compliance with the Hardy-Weinberg principle. The difference in the genotypes was significant with regards to the TG genotype when TT was used as a reference point (p = 0.03). Surprisingly, the TG heterozygous genotype provided protection [OR = 0.18, 95% CI 0.04-0.76] against cervical cancer when compared to the TT homozygous genotype. There was no significant difference in the distribution of the TT homozygous genotype in comparison with the GG homozygous state. Using the overdominant model of inheritance, TG genotype still showed lower association [OR = 0.18, 95% CI 0.05-0.69] with cervical cancer when compared to the TT and GG homozygous genotypes. 

When HPV status was considered, it was observed that TG genotype was still protective against cervical cancer among HPV positive patients (Table 4). TT and GG homozygous genotypes have the same risk for cervical cancer. Among HPV negative patients, it was shown that GG genotype has 4 times higher OR for cervical cancer compared to the TT genotype, however, the difference was not statistically significant.

## Discussion

In this study, we determine the frequency of *PIK3CA* and *MDM2 *SNP309 (*rs2279744*) in Filipino cervical cancer patients. At this stage, there is still no clear association between the presence of *PIK3CA* mutation and *MDM2 *polymorphisms and the occurrence of cervical cancer. There are few studies in this area and the conclusions remain inconclusive. These genetic mutation and polymorphisms have not been studied for cervical cancer patients in the Philippines. This was the first study on Filipino patients.

In this study, *PIK3CA* mutation (10.71%) was only found among cervical cancer patients. This is consistent with the study conducted among Chinese population where 13.6% (105 of 771) of patients with resected cervical cancer harbored non-synonymous *PIK3CA* mutations (Xiang et al., 2015). One previous study about genetic alterations in cervical cancer patients determined that *PIK3CA* has the highest gene mutation rates among all other cancer-associated genes included in their study. *PIK3CA* mutation rate was as high as 62.5% among American Indian population (Femi, 2018). Another study conducted in Italy revealed a 5% prevalence of *PIK3CA* mutations among patient with cervical squamous cell carcinoma (Tornesello et al., 2014). These studies show the possible association between *PIK3CA* mutations and the occurrence of cervical cancer. 

This study showed that E545K was the most common site of mutation in *PIK3CA* gene. This is similar to the results of previous studies that E545K is the most predominant site of mutation in the helical domain of the *PIK3CA* gene (Akbarov et al., 2017; Lou et al., 2015; Xiang et al., 2015). This missense mutation is associated with increased enzymatic activity independent of upstream signaling which results to stimulation of signalling through the Akt pathway thereby increasing cell invasion and metastasis (Arjumand et al., 2016).

HPV genotyping was previously done for all patients included in this study. This mutation was more common among HPV-negative cervical cancer samples (28.57%) than in HPV-positive cervical cancer samples (4.76%). However, the difference was not statistically significant. A previous study found no correlation between HPV infection and *PIK3CA* status. However, *PIK3CA* mutation positivity rate was relatively low among HPV-positive cervical cancer patients (Cui et al., 2009). These findings support the possibility that the *PIK3CA* mutation and HPV infection may act independently in cervical carcinogenesis.

In this study, showed that GG genotype was associated with cervical cancer regardless of the HPV status of the patient. This parallels the results of previous studies that GG genotype increases the risk of cervical cancer. It is also significantly associated with lymph node metastasis in cervical cancer (Guo et al., 2016; Jianget al., 2010; Singhal et al., 2013).

We also observed that *MDM2 *SNP309 T allele was more common that G allele in cervical cancer patients. The TT and GG homozygous genotypes have higher risk of cervical cancer compared to the TG genotype. The TG genotype seems to have a protective role against cervical cancer. However, in previous studies on cervical cancer, the G allele and TG genotype have higher risk of developing cervical cancer compared to the TT genotype. In other types of cancer such as oropharyngeal carcinoma, *MDM2 *SNP309 TT genotype has higher association with tumor recurrence when compared to TG and GG genotypes (Guo et al., 2016; Jiang et al., 2010; Singhal et al., 2013; Zhang et al., 2017).

It should be noted that there were also previous studies which showed no significant association between *MDM2 *SNP309 and cervical cancer. The allele or genotype frequency are the same between cervical cancer patients and healthy control patients (Hu et al., 2010; Meissner et al., 2007; Zhang et al., 2017). These differences regarding the role of *MDM2 *SNP309 in cervical cancer may be due to the racial differences. Hence, further studies are needed to confirm whether ethnicity can affect the distribution of *MDM2 *SNP309.

The limitations of this study include the small sample size and the inclusion of only one histological type of cervical cancer. Comparison of the different histological types of cervical cancer may also be warranted for future studies to determine whether the frequency of *PIK3CA* mutation and *MDM2 *polymorphism differ based on the histological types of cervical cancer. Future cohort studies with large sample size are needed to establish the association of these genes in cervical cancer.
